# Above-the-knee replantation in a child: a case report with a 24-year follow-up

**DOI:** 10.1007/s11751-015-0230-6

**Published:** 2015-11-12

**Authors:** Claire Marie C. Durban, Seung-Yup Lee, Hong-Chul Lim

**Affiliations:** Department of Orthopaedic Surgery, Guro Hospital, Korea University Medical Center, 80, Guro-Dong, Guro-Gu, Seoul, 152-703 Republic of Korea

**Keywords:** Lower limb, Lower limb replantation, Above-knee amputation, Replantation in children

## Abstract

Replantation of an amputated limb is generally contraindicated in crushing and traction injuries. Injury to muscle tissue and skin also creates difficulties in coverage, and bony fractures may shorten limb length which can impede lower extremity function. Numerous cases have been reported on the successful replantation of the lower limb in children; however, review of previous English literature has documented only very few replantation at the thigh level, and those with severe crushing injury resulted in subsequent amputation. We report a case of successful thigh-level replantation in a 3-year-old child who sustained a crushing–traction type of injury with a follow-up of 24 years. After the replantation, early and late complications developed but these were successfully managed. On her last visit, the patient had pain-free ambulation without assistance, had intact protective sensation distal to the injury, and was very satisfied with the outcome. Replantation of the lower limb in children with crushing or avulsion type of injuries is still a worthwhile procedure. However, both the patient and the family should be aware that multiple surgeries may be needed to accommodate to long-term complications such as joint stiffness, scar contractures, and limb length discrepancies.

## Introduction

Replantation of an amputated limb is contraindicated in crushing and traction injuries generally because injury to the nerves and vessels is misleading when compared to the actual zone of injury [1, 2]. Injury to muscle and skin will create difficulties in cover, and fractures which shorten limb length impede function. Unlike the upper limb, criteria for replantation of the lower extremity do not exist [3].

Numerous cases have been reported on the successful replantation of the lower limb in children [3–10] with few reporting long-term functional outcomes [3, 5, 9]. There are very few replantations at the thigh level [2, 10, 11]. There are no reports in the literature which document successful replantation of the lower extremity in a child from a traumatic amputation above the knee associated with degloving through a pneumatic tire injury. We report a case of successful lower extremity replantation in a child with such a complex soft tissue injury.

## Case report

**Fig. 1:**
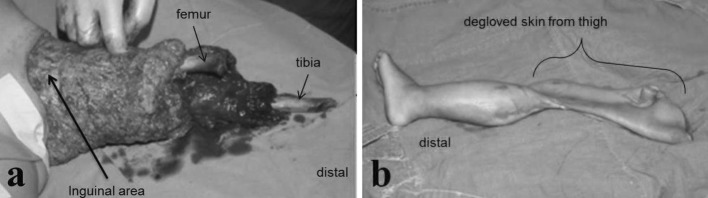
Initial injury of the patient showed **a** complete degloving of the thigh up to inguinal area and a fracture through the distal femur; knee and proximal tibia are partially attached to the lateral portion of the thigh. **b** Skin is intact from the foot up to the proximal thigh

On April 23, 1986, a 3-year-old girl was brought to our institution after a 1.5-ton truck ran over her left lower limb. She suffered a floating knee injury with fractures at the supracondylar level of the femur and middle third of the tibia. An above the knee and near-total amputation was sustained at the supracondylar level, with a complete degloving of the thigh up to the inguinal area (Fig. 1). There were no other injuries.

Replantation started at 9 h after injury. The supracondylar fracture was fixed with Kirschner wires, followed by intramedullary nailing of the tibial fracture using a Steinmann pin. The popliteal artery was anastomosed using a 5-cm vein graft from the contralateral saphenous vein after which two popliteal vein anastomoses were performed. The peroneal and tibial nerves were then repaired followed by repair of the quadriceps femoris, hamstrings and triceps surae. The degloved skin was defatted and was re-applied as a full-thickness skin graft to the thigh.

**Fig. 2:**
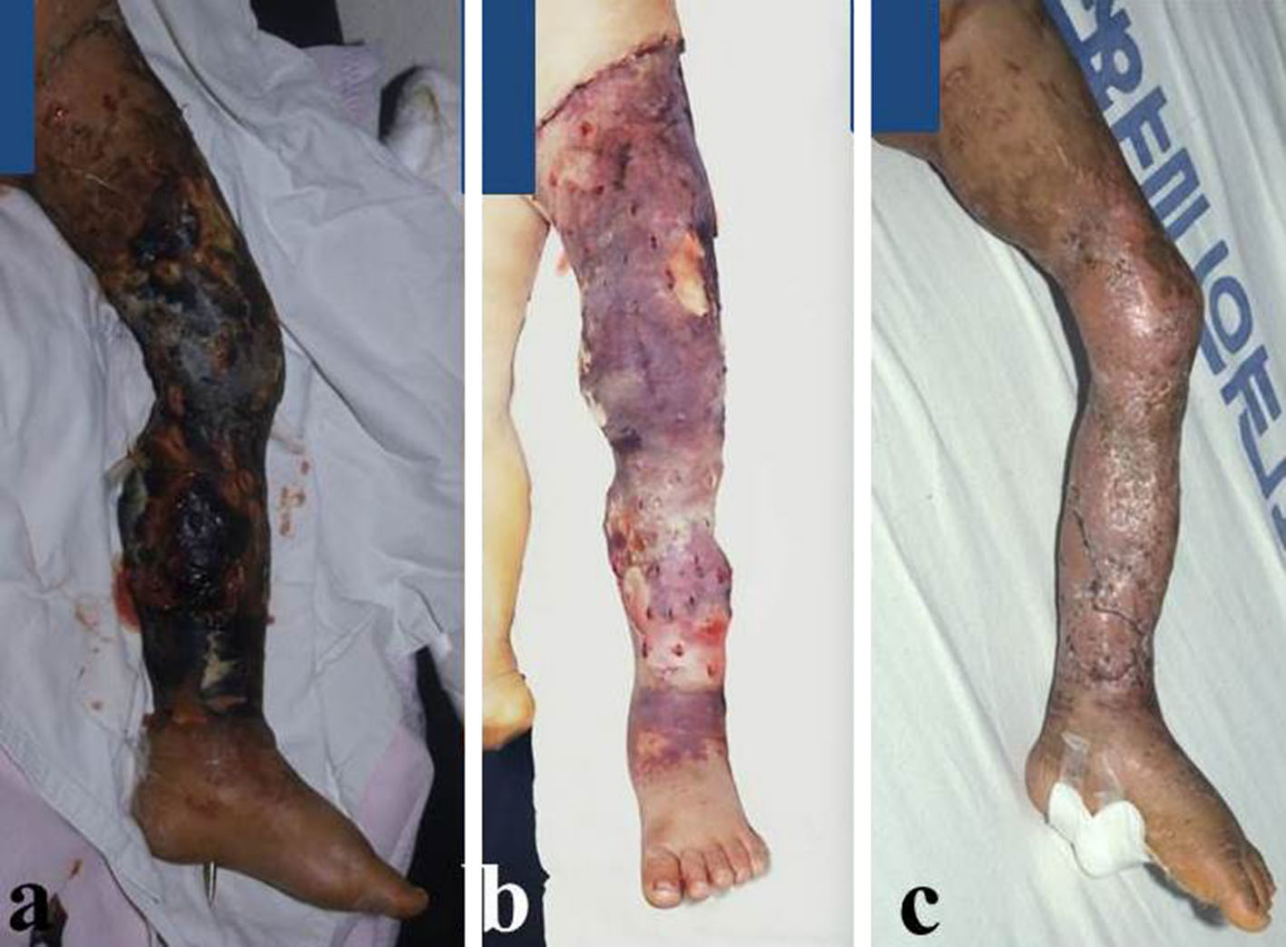
Skin and muscle necrosis **a** evolved a few days after index surgery; STSG (split-thickness skin graft) was applied over the entire lower limb, **b** after multiple debridement, which proceeded to heal, **c** without further intervention

The patient was stable postoperatively. A few days after surgery, skin and muscle necrosis of the gastroc-soleus complex developed, requiring repeated debridement before a split-thickness skin graft could be applied (Fig. 2). Two months after the index surgery, the patient had begun rehabilitation exercises for the lower extremity. After removal of the Steinman pin, an anteromedial external fixator was applied for two and a half months on the tibia in order to facilitate range of motion and weight-bearing exercises. The patient was discharged 1 month later.

**Fig. 3:**
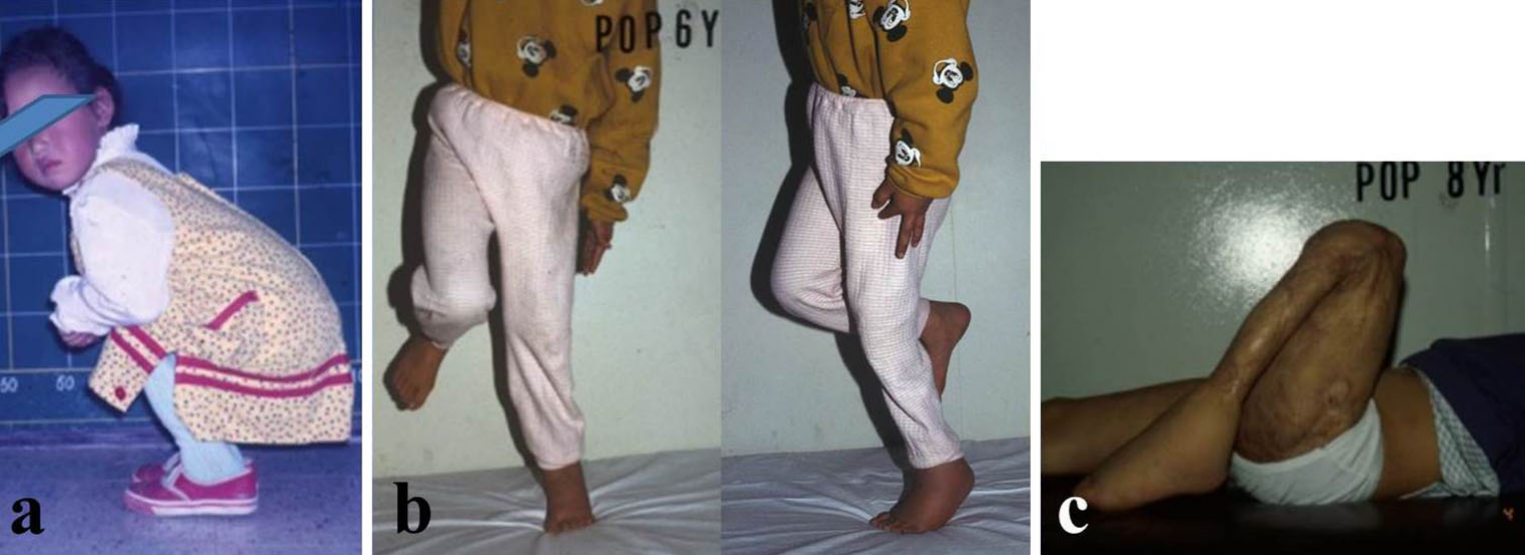
Serial follow-up beginning 4 years (**a**), 6 years (**b**), and 8 years (**c**) after surgery demonstrating worsening knee and ankle contractures

At 4 years after the injury, the patient was ambulatory without assistance and no leg length discrepancy was noted at the time; knee flexion was noted at more than 90° and ankle dorsiflexion was to 10°. Six years after injury, the patient was able to stand on the affected limb without support, but soft tissue contractures had developed in the ankle and knee joints. No intervention was done at this time. When patient was 11 years old, 8 years after the injury, the knee flexion contracture was severe and a Z-plasty of the popliteal area to release the scar tissue contracture (Fig. 3) was carried out. With continued rehabilitation, the patient was able to regain full knee extension though flexion was limited to 90°. She was ambulatory without support but left with an equinus deformity of the left ankle. The leg length discrepancy had become more pronounced at this time of a growth spurt. She underwent her last major surgery when she was 12 years old; a femoral osteotomy was done, and an external fixator was applied for gradual lengthening to correct the 6-cm shortening of the left lower extremity.

**Fig. 4:**
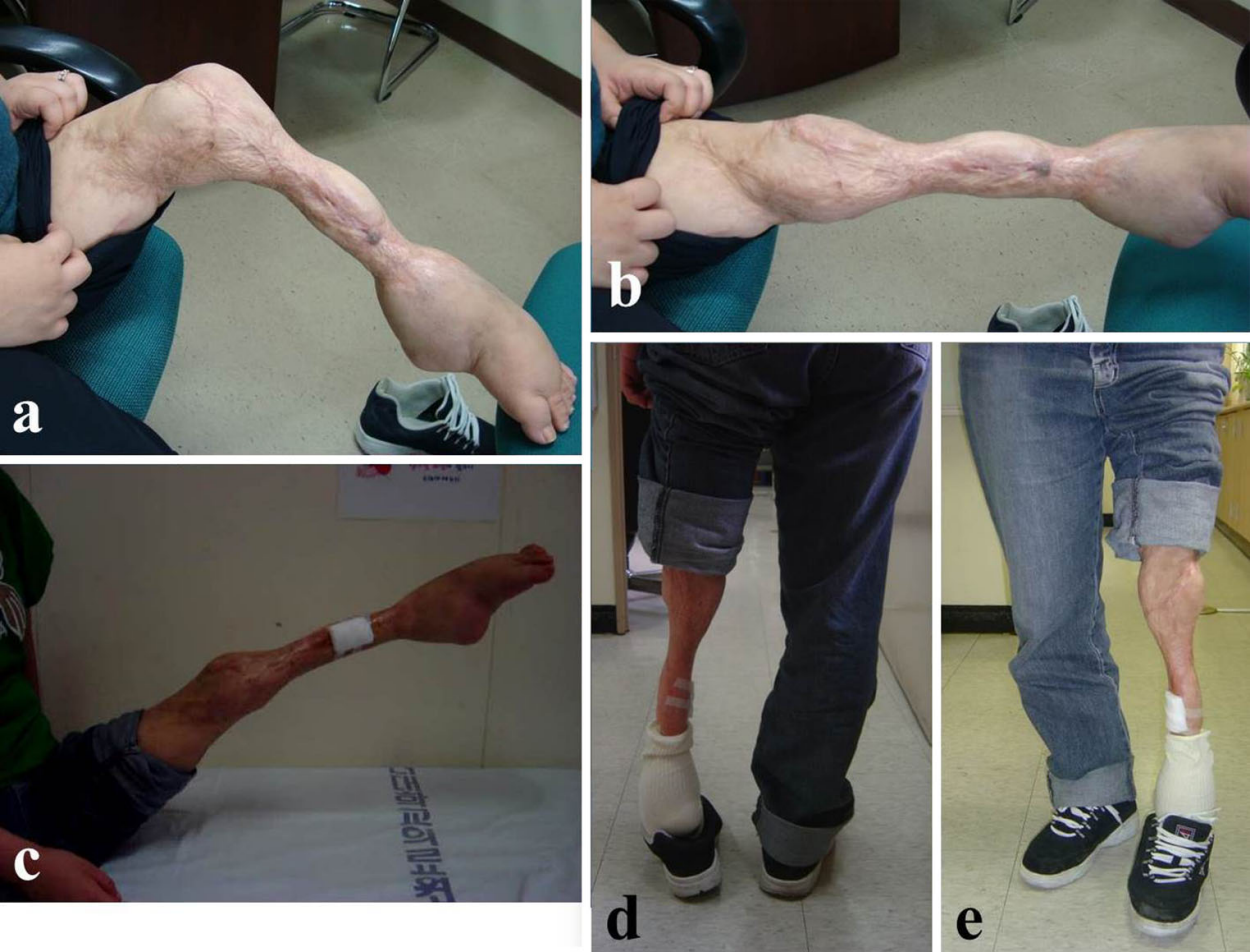
On last visit, knee flexion was 90° (**a**), extension was full (**b**, **c**); patient ambulated with an equinus deformity (**d**, **e**)

**Fig. 5:**
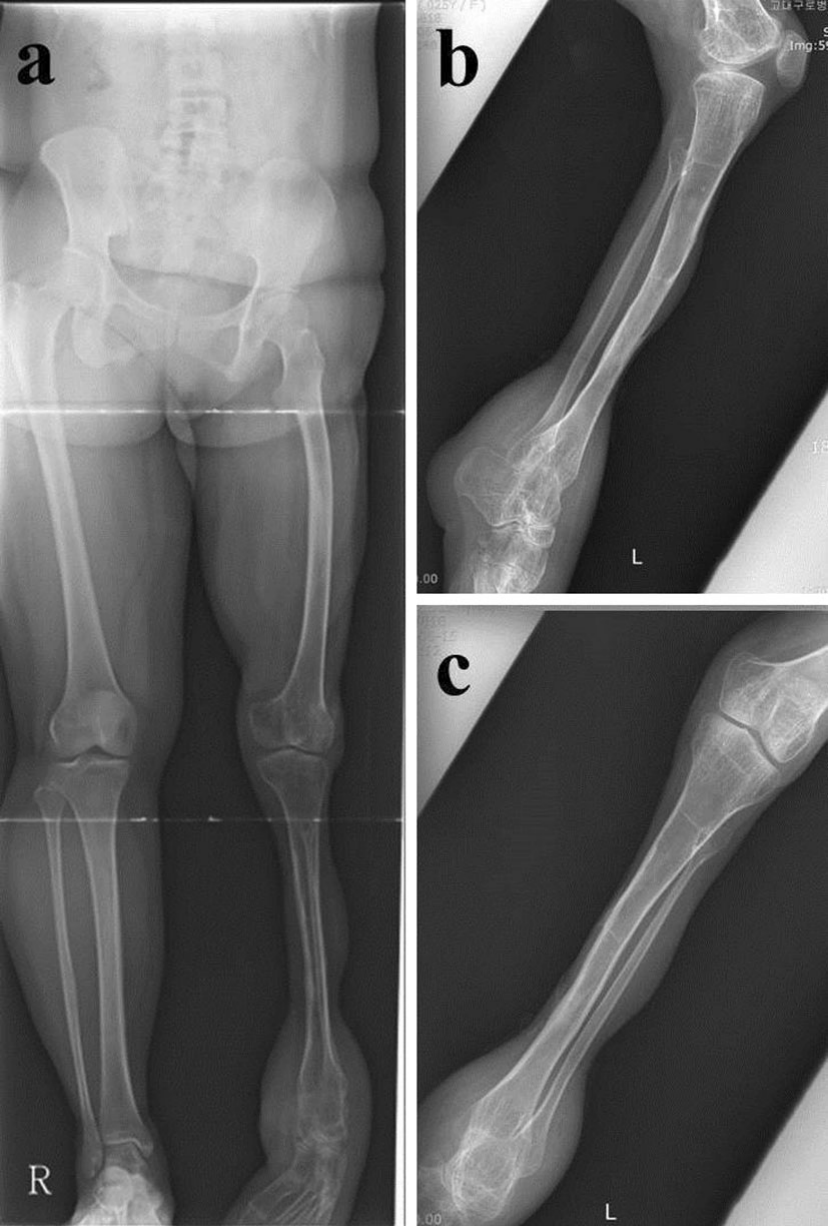
Standing roentgenogram shows pelvic tilting and shortened left lower extremity (**a**); tibia had remodeled completely (**b**, **c**)

At the last review in September 2009, the patient was 25 years old. At 21 years after the incident, she remained freely ambulatory but with an equinus deformity of the left ankle. The knee range of motion was maintained at 0–90 degrees of flexion, the foot was sensate, and she was also able to move her toes (Fig. 4). Radiographs taken showed left lower extremity was shorter but the fractures and osteotomy sites had completely remodeled (Fig. 5). In 2012 through a telephone interview, the patient described her knee movement as unchanged and had no new complaints. She was satisfied with the functional outcome of her left lower limb despite it not being esthetically pleasing. Aside from the operative scars from the skin grafts, the limb was thinner from severe reduction in muscle bulk over the leg where debridement had been done. The patient was content that she was walking with her own limb with intact sensation and had avoided a prosthesis.

## Discussion

Decision making in traumatic amputations is difficult, especially for young children. Multiple scoring systems have been proposed to guide the management of complex extremity trauma, but none are helpful. Reports of results after replantations of limbs from thigh-level amputations are not promising: One patient described by Battiston et al. [2] who sustained a crushing injury to the thigh, despite initial replantation, had an amputation later due to postoperative hemorrhage; Gao et al. [11] reported a 19-year-old patient who did not regain plantar protective sensation after replantation and suffered severe cold intolerance. This painful and dysfunctional lower limb was subsequently amputated. Although this patient was young, extensive injury to the sciatic nerve from crush may have been responsible to poor recovery of limb sensibility. Despite these discouraging results, replantation was carried out because this patient was very young and it was felt limb preservation should be attempted. Previous studies have demonstrated the outstanding healing potential of children who sustained amputations at different levels [3–5, 7–10, 12]. Datiashvili [5, 9] reported a 3-year-old child with successful replantation of both lower limbs. Initial leg length and foot length discrepancies were corrected during the first year after surgery, and both lower extremities and feet grew symmetrically thereafter. Sensation was re-established over the distal legs as early as 2 months postoperatively. Subsequent follow-up reports documented the patient was ambulatory and fully satisfied with the outcome. Bajec and Srakar [7] also reported replantation for a bilateral amputation in a 2-year-old boy. Although the outcome was ultimately successful, the patient had 5 further interventions for correction of leg length discrepancy and scar-related complications. Masuda et al. [8] reported a 17-year follow-up of a 4-year-old child who underwent replantation of the distal leg. The child recovered ankle range of motion and sensation distal to the injury site with the leg length discrepancy improving with time.

This case represents the longest follow-up period (23 years) for a lower limb replantation in a child. Despite the fact that debridement of all grossly non-viable tissue was done during the index surgery, postoperative necrosis of the triceps surae muscles ensued. A probable reason is the injury being combined crushing and traction trauma: The zone of injury is more extensive than that visible in crushing and avulsion type of amputations; secondly, muscle necrosis from pneumatic injuries evolves hours to days after the incident and may account for the calf muscles appearing viable during the initial debridement; and thirdly, the warm ischemia time was greater than 9 h before reperfusion achieved. In contrast, in the upper limb in proximal amputations (where there is greater muscle bulk), warm ischemia time is limited to 6 h only [13].

Radical debridement of all non-viable and crushed tissue is essential to limb survival. This leads to considerable shortening of the replanted leg and to a reduction in function, aside from the cosmetic problem of a scarred and muscle-deficient extremity. Bone and soft tissue injuries around the joints may cause joint stiffness and contractures, with the latter needing surgical release to regain a functional range of motion. Large nerve and tendon gaps preclude primary repair and may require staged reconstructions. These conditions are expected but should not discourage the surgeon from performing replantation if other variables are favorable. It is imperative that the family and patient are advised regarding the various sequelae after replant surgery and the treatment options for each. A long-term treatment plan should be tailored for these patients for timely management of late complications. The ultimate goal of the replant surgery is to provide a non-painful, functional limb and not just a viable one.

## Conclusion

Replantation of the lower limb in children with crushing or avulsion type of injuries is a worthwhile procedure. Expectations have to be realistic over the final limb function, appearance, and multiple surgeries to manage long-term complications such as joint stiffness, scar contractures, and limb length discrepancy.
